# Host Cell Signalling and *Leishmania* Mechanisms of Evasion

**DOI:** 10.1155/2012/819512

**Published:** 2011-11-03

**Authors:** Marina Tiemi Shio, Kasra Hassani, Amandine Isnard, Benjamin Ralph, Irazu Contreras, Maria Adelaida Gomez, Issa Abu-Dayyeh, Martin Olivier

**Affiliations:** Centre for the Study of Host Resistance, Research Institute of the McGill University Health Centre and Departments of Microbiology and Immunology and Medicine, McGill University, Room 610, 3775 University Street, Duff Medical Building, Montréal, QC, Canada H3A2B4

## Abstract

*Leishmania* parasites are able to secure their survival and propagation within their host by altering signalling pathways involved in the ability of macrophages to kill pathogens or to engage adaptive immune system. An important step in this immune evasion process is the activation of host protein tyrosine phosphatase SHP-1 by *Leishmania*. SHP-1 has been shown to directly inactivate JAK2 and Erk1/2 and to play a role in the negative regulation of several transcription factors involved in macrophage activation. These signalling alterations contribute to the inactivation of critical macrophage functions (e.g., Nitric oxide, IL-12, and TNF-**α**). Additionally, to interfere with IFN-**γ** receptor signalling, *Leishmania* also alters several LPS-mediated responses. Recent findings from our laboratory revealed a pivotal role for SHP-1 in the inhibition of TLR-induced macrophage activation through binding to and inactivating IL-1-receptor-associated kinase 1 (IRAK-1). Furthermore, we identified the binding site as an evolutionarily conserved ITIM-like motif, which we named kinase tyrosine-based inhibitory motif (KTIM). Collectively, a better understanding of the evasion mechanisms utilized by *Leishmania* parasite could help to develop more efficient antileishmanial therapies in the near future.

## 1. Background

Apart from the impact of *Leishmania* on world health, Leishmaniasis represents an elegant infection model that can teach us a lot about host-parasite interactions and immune evasion. This parasite has the ability to enter host macrophages (M*∅*s) safely and replicate inside the very same phagocytes that were recruited to destroy it. The inability of M*∅*s to kill the parasite and activate cells of the adaptive immune system is a product of the parasite's long-reported capacity to alter several key signalling pathways in the host. Many signalling alterations are seen early in the course of infection suggesting they start upon the initial contact between the parasite and the M*∅*. These rapid alterations of signalling pathways serve at least two main functions: firstly, inhibition of M*∅* killing mechanisms that are triggered upon phagocytosis of foreign particles (e.g., production of reactive oxygen species), and secondly, inhibition of leishmanicidal functions that can be triggered in response to M*∅* activation in infected tissues in response to stimuli such as lipopolysaccharides (LPS) or interferon-*γ* (IFN-*γ*) (e.g., nitric oxide production). In this review, we will discuss the roles of *Leishmania* in disease establishment, focusing on the signalling pathways that they interfere with and the M*∅* functions that are affected by the alteration of these pathways.

## 2. Alteration of Macrophage Signalling Molecules by *Leishmania*

Several pathogens (i.e., *Acanthocheilonema viteae* [1], African trypanosomes [2], and *Toxoplasma gondii* [3]) are able to alter the signalling of their target cells to their own advantage and *Leishmania* is no exception. *Leishmania* achieves this by either employing strategies to inhibit proteins that play a positive role in immune cell activation or by activating molecules known to play key roles in the negative regulation of immune cell signalling and function [4]. We will discuss below the main signalling molecules altered by *Leishmania* in an effort of the parasite to survive inside host M*∅*s. 

Firstly, protein kinase C (PKC), a protein family comprising 10 serine/threonine kinases, initially characterized as Ca^+2^ and phospholipid dependent [5, 6], is classified into three subfamilies: the conventional (PKC-*α*, -*β*I, -*β*II, and -*γ*), novel (PKC-*δ*, -*ε*, -*η*, and -*τ*), and atypical (PKC-*ζ* and -*λ*) isoforms [7]. PKC signalling is known to play a key role in the regulation of M*∅* functions activating, for instance, cytokines such as IFN-*γ* and TNF-*α* [8, 9], both having important roles in driving several M*∅* functions including NO production [8] and oxidative burst [10]. Promastigote LPG has been described to be able to block PKC activity [11–13]. This inhibition is achieved through the binding of LPG to the regulatory domain of PKC which contains the DAG, Ca^+2^, and phospholipid binding sites [14]. It is interesting to observe that amastigotes, which lack LPG, are also able to inhibit PKC activity in monocytes [15], suggesting that factors other than LPG can also mediate this inhibitory effect. Indeed, *Leishmania*-induced ceramide generation [16] and GIPLs [12] have been shown to be able to do so, providing a possible mechanism whereby amastigotes can inhibit PKC activity. 

Later on, Janus kinase 2 (JAK2) as one of four members of the Janus family of tyrosine kinases (JAK1, JAK2, JAK3, and TYK2), has been identified to be importantly affected by *Leishmania* infection. JAK activation plays an important role in cell proliferation, differentiation, migration, apoptosis, and immune activation [17]. The JAK signalling pathway is initiated when a cytokine or a growth factor binds to its receptor inducing receptor multimerization followed by JAKs transphosphorylation and activation, ultimately leading to the phosphorylation of signal transducer and activator of transcription (STAT), a transcription factor (TF), that will then dimerize and proceed to nucleus by translocation and to bind target regulatory sequences to activate or repress transcription [17, 18].

**Figure 1:**
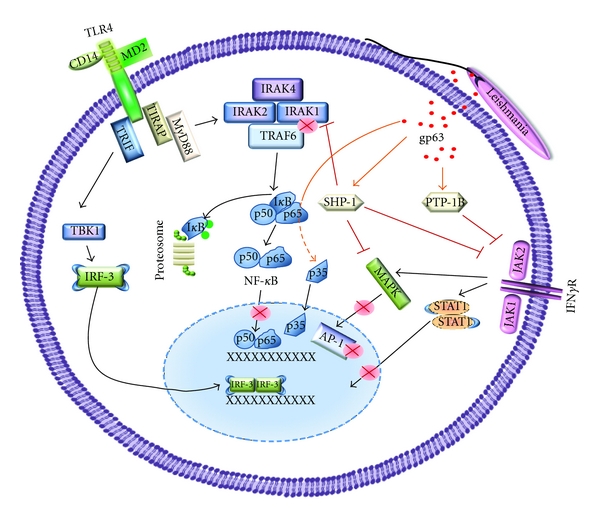
*Downregulation of macrophages signalling by Leishmania infection. Leishmania* infection modulates phosphatases (SHP-1 and PTP-1B) activity by mechanism involving the metalloprotease gp63. SHP-1 was found to interact with IRAK-1, a key kinase involved on TLR-triggered signaling pathway. Whereas, both SHP-1 and PTP-1B are involved in the downregulation of IFN*γ*-induced pathway (JAK/STAT1) as well as MAPK activation. In addition, transcription factor such as NF-*κ*B and AP-1 are cleaved/degraded in part by *Leishmania* gp63. Orange arrows indicate gp63 involved modulation; black arrows indicate activation; red abrogated lines indicate downregulation.

Importantly, the iNOS gene promoter responsible for NO production has binding sites for several TFs including STAT-1 [19, 20]. *Leishmania* has the ability to block the JAK/STAT signalling pathway in response to IFN-*γ* stimulation, therefore avoiding the induction of NO. Indeed, it has been reported that infection with *L. donovani* amastigotes was able to block IFN-*γ*-induced JAK1, JAK2, and STAT-1 phosphorylation in PMA-differentiated U-937 promonocytic cells and human monocytes [21]. However, we went further in studying the effect of *Leishmania* on JAK2 phosphorylation by reporting that *L. donovani* promastigotes were rapidly activating host SHP-1 leading to the subsequent inhibition of IFN-*λ*-induced JAK2 phosphorylation [22], see Figure 1. Others have suggested that IFN-*γ* unresponsiveness to stimulation can be due to the inhibition of the IFN-*γ* receptor (IFN-*γ*R) complex formation [23], however they did not provide any clues on how the parasite could do so. Another complication with this report is that the authors infected cells for 24 hours to see an appreciable effect on receptor expression and phosphorylation, which cannot explain the rapid dephosphorylation of JAK2 seen when BMDMs are infected with *Leishmania* promastigotes [22], supporting the notion that early JAK/STAT inhibition must depend on parasite-induced alterations of existing signalling molecules of the host and not on alterations at the transcriptional level.

In the same line of ideas, several members of the mitogen-activated protein kinases (MAPKs) family (e.g., extracellular signal-regulated kinase1/2 (Erk1/2), proline for Jun N-terminal kinase (JNK), and glycine for p38), known to play critical role in the activation of several TFs [24], have been found to be exploited by *Leishmania* parasite (Figure 1). Indeed, as was the case with the JAK family, it is remarkable, though not unexpected, that the *Leishmania* parasite developed tactics to render several MAPK members inactive in response to parasite entry to M*∅*s or to activating stimuli that follow infection. For instance, it was reported that the phagocytosis of *L. donovani* promastigotes by naive M*∅*s does not lead to the activation of any of the three MAPKs (Erk1/2, JNK, p38) [25]. Furthermore, activation of several MAPKs in response to LPS has been shown to be inhibited in infected cells. For instance, *L. amazonensis* amastigotes are able to block LPS-mediated Erk1 phosphorylation in infected M*∅*s [26], and *L. donovani* amastigotes can block PMA-induced Erk1/2 phosphorylation in RAW264 M*∅*s leading to the inhibition of Elk-1 and c-fos expression [27]. The authors of the latter study suggested a role for host PTPs in Erk1/2 inactivation, a hypothesis supported and more deeply explored by our laboratory where we provided evidence that PTP-SHP-1 is able to dephosphorylate and inactivate Erk1/2 through demonstrating that this MAPK was still able to be activated in *Leishmania*-infected SHP-1-deficient M*∅*s in response to IFN-*γ* stimulation [28]. Alternatively, it has been suggested that ceramide production by *L. donovani*-infected M*∅*s can lead to reduce Erk1/2 phosphorylation [29]. Interestingly, amastigotes of *L. mexicana* were also reported to inhibit Erk1/2 signalling not by inhibiting their phosphorylation, but rather by degrading them using the parasite's cysteine proteinases. Similar cysteine proteinase-dependent degradation was observed for JNK [30].

In regard to p38, it has been shown that this MAPK is nonresponsive when M*∅*s infected with *L. major* are stimulated with a CD40 antibody to mimic the M*∅*-T cell interaction. p38 inactivation correlated with impaired iNOS2 expression and NO production and therefore impaired leishmanicidal functions [31]. In fact, this inactivation makes sense in the light of experiments showing the importance of p38 activation in the control of *Leishmania* infection. The use of anisomycin, a p38 activator, enhanced parasite killing in M*∅*s by triggering p38-dependent antileishmanial effects [31, 32].

In order to inhibit gene expression of proinflammatory cytokines and microbicidal molecules, *Leishmania* developed several strategies to interfere with TFs that bind to the promoters of those genes. Several TFs are involved in this process including NF-*κ*B, STAT-1*α*, and AP-1, all of which known to be modulated by the parasite. In fact, several groups have reported different strategies employed by *Leishmania* to alter, for instance, the TF NF-*κ*B. *Leishmania*-induced ceramide generation by M*∅*s was shown to play a role in NF-*κ*B inhibition [29]. One study provided evidence that *L. major* amastigotes blocked the nuclear translocation of the p65/p50 complex selectively favouring the c-Rel/p50 complex that, they proposed, plays a role in the gene expression of immunosuppressive cytokines in M*∅*s such as IL-10 [33]. Another study reported that cysteine proteinases of *L. mexicana* mediated NF-*κ*B degradation and caused its inability to bind its DNA consensus sequence, thus partially explaining how the parasite can inhibit LPS-mediated IL-12 production [30]. Work from our laboratory showed that promastigotes of several pathogenic *Leishmania* species were able to cleave the p65 RelA subunit to generate a p35 RelA fragment translocating to the nucleus and bind DNA. This p35 fragment was suggested to be involved in the parasite's ability to drive NF-*κ*B-mediated chemokine gene expression in infected M*∅*s [34].

In addition to NF-*κ*B and the fact we have previously described *Leishmania*'s ability to inhibit the JAK/STAT pathway [22], our laboratory has also reported that the parasite is able to repress IFN-*γ*-mediated signalling in M*∅*s by interfering with STATs. We showed that *L. donovani* promastigotes were able to cause proteasome-mediated STAT-1 degradation in infected M*∅*s. However, whereas STAT-1 degradation was reversed using proteasome inhibitors [35], its capacity to respond to IFN-*γ* was still altered due to JAK2 inactivation (unpublished data).

AP-1 is a structurally complex TF formed by dimmers from Jun and Fos protein family [36] that can be activated by many kinds of stimuli such as growth factors, cytokines, hormones, and pathogens, which do so using several signalling molecules. Consequently, the previously mentioned tactics employed by *Leishmania* to interfere with PKC, Erk1/2, JNK, and p38 activities have a direct impact on the ability of the parasite to block AP-1 signalling in M*∅*s. Importantly, work from our group demonstrated a role for SHP-1 in AP-1 inhibition [28, 37] and, more recently, that the parasite's surface protease gp63 is responsible for the cleavage and degradation of key AP-1 subunits [38]. The latter finding provides the first demonstration that a parasite-derived molecule can directly interfere with AP-1 in host M*∅*s in order to block its downstream functions.

## 3. Negative Regulation by Protein Tyrosine Phosphatases

Protein tyrosine phosphatases (PTPs) are proteins that have the ability to dephosphorylate substrates and are divided into receptor-like and nonreceptor PTPs. Nonreceptor PTPs can either dephosphorylate tyrosines only or can possess dual specificity dephosphorylating tyrosines as well as serines/threonines [39]. One common feature of PTPs is the presence of a PTPs catalytic domain in which a critical cysteine is found within a conserved signature motif, (I/V)HCxxGxxR(S/T), and mediates the hydrolysis via the formation of a thiophosphate intermediate [40]. Receptor-like PTPs include RPTP-*α*, CD45, and CD148, and the functions of some like CD45 in immune cell signalling are well known [41]. However, herein we will focus on a selected group of soluble PTPs that have been shown to play a role in *Leishmania* host evasion mechanisms, namely, PTP-1B, TC-PTP, PTP-PEST, and most importantly SHP-1. PTP-1B and TC (T cell)-PTP: they are two ubiquitously expressed PTPs that have more than 73% identity in their catalytic domain [42]. PTP-1B is known to play important regulatory functions in metabolism, as demonstrated by the insulin hypersensitivity of PTP-1B^−/−^ mice and their resistance to high-fat-diet-induced obesity [43, 44]. This insulin hypersensitivity was shown to be due to the ability of PTP-1B to dephosphorylate the insulin receptor [45]. PTP-1B also seems to play a role in the regulation of cytokine signalling through its ability to interact with and dephosphorylate members of the JAK family, namely, JAK2 and TYK2 [46]. In addition to PTP-1B's role in the regulation of JAK/STAT signalling, a role for this phosphatase in the regulation of TLR4 signalling was proposed. PTP-1B^−/−^ M*∅*s had increased LPS-induced iNOS expression and NO production compared to WT M*∅*s and were more susceptible to endotoxic shock following low-dose LPS injection [41]. As PTP-1B was found to be an important negative regulator of M*∅*s signaling, its role in Leishmaniasis could be critical. In fact, we recently showed that *Leishmania* gp63 was able to enhance PTP-1B activation by cleaving it. PTP-1B activity seems to inhibit M*∅* activation and help in parasite survival as seen in the delayed onset of footpad swelling and reduced parasite burden in PTP-1B^−/−^ mice infected with *L. major* [47]. In addition, we found that TC-PTP that also plays important roles in the negative regulation of JAK1, JAK3 [48], and nuclear STAT-1 [49] was modulated by gp63 in *Leishmania*-infected M*∅*s [47]. This gp63-mediated TC-PTP cleavage along with the cleavage of PTP-PEST was recently reported by our group and M.L. Tremblay's group to enhance the catalytic activity of the PTPs in question and/or allow them to access additional substrates that might help the parasite establish itself [50].

Another PTP modulated by *Leishmania* gp63 is SHP-1. This PTP contains two N-terminal SH2 domains (N-SH2, C-SH2), followed by a PTP domain responsible for dephosphorylating substrates, and a C-terminal tail [41]. This phosphatase is mostly expressed in hematopoietic cells [51, 52], but is also expressed at lower levels in epithelial [52], endothelial [53, 54], and central nervous system cells [55]. The SH2 domains have two main functions: firstly, the N-SH2 domain plays an important autoinhibitory role by interacting intramolecularly with the PTP domain, keeping the PTP in the inactive state. Secondly, both SH2 domains have the ability to bind to phosphotyrosine (p-Y) residues usually found within immunoreceptor tyrosine-based inhibitory motifs (ITIMs) whose consensus sequence is (I/V/L/S)xYxx(L/V) [36]. This second feature of SH2-domains is thought to play a role in the detachment of the N-SH2 from the PTP domain once the C-SH2 domain binds to a target p-Y, therefore opening up and activating the PTP [41].

At the signalling level, our laboratory have clearly demonstrated that *Leishmania* was able to rapidly activate host SHP-1 causing SHP-1-mediated JAK2 inactivation in M*∅*s [22]. Additionally, we and others have implicated SHP-1 in the negative regulation of Erk1/2 activity [27, 28] and in the regulation of the downstream TFs NF-*κ*B and AP-1 [28] during *Leishmania* infection. At the functional level, our laboratory showed that the injection of PTP inhibitors (bpV-phen, a bis-peroxovanadium compounds) to mice infected with *L. major *or *L. donovani* helped control the infection [56] in a manner dependent on iNOS expression and NO production [57]. Furthermore, we demonstrated that SHP-1-deficient viable moth-eaten mice infected with *L. major* did not develop footpad swelling and had significantly reduced parasitic loads [58]. This decreased pathology was associated with increase activated neutrophil recruitment to the footpad and more iNOS mRNA expression [58]. 

As to how *Leishmania *is able to activate SHP-1, it has been proposed that *Leishmania*'s elongation factor-1*α* (EF-1*α*) is responsible for the activation of host SHP-1 seen 16 hours after infection [59]. This report cannot explain, however, how SHP-1 is activated in earlier infection times nor does it explain how EF-1*α* of the parasite can shuttle from the phagolysosome where the parasite is to the cytosol where SHP-1 is found. A more plausible mechanism has been recently suggested by our group, where SHP-1 was shown to be activated via cleavage by the parasite's protease gp63, which gains access to the cytosol by going through the lipid raft of host M*∅*s [47]. 

Collectively, it appears that the rapid activation of SHP-1 by *Leishmania *is a key host evasion step whereby the parasite is able to utilize this phosphatase to negatively regulate several key M*∅* pathways and render them unresponsive to activating stimuli such as IFN-*γ* and LPS. By doing so, the parasite is able to block several M*∅* functions such as NO production and the synthesis of many proinflammatory cytokines that can be deadly to the parasite if allowed to be produced.

## 4. Macrophage Functions Altered by *Leishmania*

Modulation of signalling pathways by *Leishmania *is intended to alter critical M*∅* functions to the advantage of the parasite. Upon the initial contact of *Leishmania *with the M*∅*, certain functions such as the production of chemokines and chemokine receptors are induced, whereas others are inhibited. Among the functions inhibited by the parasite are those related to M*∅* activation and to their ability to present Ag and communicate with cells of the adaptive immune system. Hereby, we will discuss the main functions that *Leishmania* can interfere with initial interaction (0–6 h) or chronic infection (>6 h) of host M*∅*s.

One of the important early challenges confronted by *Leishmania* is the ability to preferentially recruit cells of the immune system to the site of inoculation in order to infect them and establish disease in the host without getting killed. One key mechanism by which the parasite is able to do so is the induction of chemokine expression and production by host immune cells. One study showed that infection of mice with *L. major *upregulated the gene expression of several chemokines (RANTES/CCL5, MIP-1*α*/CCL3, IP-10/CXCL10, and MCP-1/CCL2) in cells collected from the footpad and their draining lymph nodes [60]. Additionally, we have shown that *L. major *infection caused an upregulation in the expression of several chemokines (RANTES, MIP-1*α*, MIP-1*β*/CCL4, IP-10, MCP-1, and MIP-2/CXCL1) in cells being rapidly recruited at the site of inoculation [61]. It is interesting to see that most of these chemokines are monocyte chemoattractants, recruiting M*∅*s to infected tissues and helping the parasite get installed. It is equally interesting to see that none of these chemokines, with the exception of MIP-2, attract neutrophils. This is in accordance with our previous finding that exacerbated neutrophil recruitment to infection sites is associated with parasite killing in SHP-1 deficient viable moth-eaten mice [58]. 

So far, we have considered chemokine upregulation as beneficial to the parasite, yet it is important to bear in mind that secreted chemokines during Leishmaniasis can act as a double-edged sword. Whereas selective activation of chemotactic factors can help the parasite to recruit M*∅*s and neutrophils that they can infect and/or utilize during the initial step of infection, treatment of susceptible BALB/c with recombinant IP-10 in the early course of *L. major* infection has been shown to increase NK cell cytotoxic activity in the draining lymph nodes and to drive a healing IFN-*γ*-mediated Th1 response [62]. In chronic infections, chemokine types, amounts, and duration of chemotactic effect have been implicated in parasite clearance or persistence. For instance, in visceral Leishmaniasis, clearance of parasites from the liver is strongly associated with increased late phase IP-10 production and the Th1 effects associated with its presence [62]. Parasite persistence in the spleen, on the other hand, has been correlated with sustained MCP-1, but not IP-10 levels [63].

Whereas *Leishmania* can modulate selected chemokines, importantly the inhibition of key microbicidal functions is crucial for its initial survival. For instance, one of the dangers that* Leishmania* encounters recruiting and entering M*∅*s is the ability of these cells to produce deadly free radicals such as NO [64] and reactive oxygen intermediates (ROIs) [65]. NO is produced by NOS which converts one of the terminal nitrogens of the guanidino group of L-arginine to NO producing L-citrulline [66, 67]. The importance of this free radical in Leishmaniasis was demonstrated by several groups. An early study showed the ability of activated M*∅*s to kill* L. major *amastigotes by an L-arginine-dependent mechanism [68]. Later confirmed by the observation that L-N-monomethyl arginine (L-NMMA), an L-arginine analogue and inhibitor of the NO pathway was able to inhibit the leishmanicidal effect of M*∅*s activated* in vitro *with IFN-*γ* or LPS. They also showed the ability of NO in cell-free suspensions to kill the parasite. Importantly, the same group demonstrated the importance of NO* in vivo* by rendering resistant CBA mice susceptible to* L. major *infection upon local administration of L-NMMA [64].

However, parasite is able to block its synthesis in response to stimuli such as IFN-*γ* [69], but how can *Leishmania* achieve this inhibition? A critical role for host SHP-1 has been proposed. As previously stated, *Leishmania* has the ability to rapidly activate SHP-1 in infected M*∅*s and by doing so can interfere with several molecules involved in NO production including JAK2, Erk1/2, and the TFs NF-*κ*B and AP-1. Indeed, SHP-1 deficient M*∅*s infected with *L. donovani* are still able to produce NO in response to IFN-*γ* stimulation, unlike infected WT M*∅*s which are refractory to a similar stimulation [28]. As expected, the IFN-*γ*-mediated NO production in infected SHP-1 deficient M*∅*s correlated with successful JAK2 and Erk1/2 phosphorylation and the activation of NF-*κ*B and AP-1. These findings further elucidate the role of SHP-1 activation in parasite survival and propagation through its ability to contribute to NO inhibition [28]. Another mechanism involved in the downregulation of NO production is by conversion of arginine to ornithine and urea via the arginase pathway [70]. Supporting this mechanism, recently, it has been shown that arginase as well as polyamines gene expression are upregulated by *L. amazonensis* amastigote [71]. Whereas those latter mechanisms of evasion are to be of general use by all, *Leishmania* parasite will need further investigation.

In addition to NO, ROIs represent another source of danger to *Leishmania*. These intermediates include the superoxide radical and hydrogen peroxide produced by cells of the immune system such as neutrophils and M*∅*s in response to various agonists. Although important in parasite killing, the activity of the respiratory burst in mice was shown to have an early and transient effect only. This conclusion is based on the delayed granuloma formation and resolution of infection seen in respiratory burst-deficient X-CGD mice infected with *L. donovani* compared to WT [72]. Despite the critical role that NO seems to play in *Leishmania* killing [72], ROIs do contribute to parasite clearance and are therefore a target to be inhibited by the parasite. Indeed, *L. donovani* has been shown to inhibit the oxidative burst in infected M*∅*s [15, 73, 74], and this inhibition was in part mediated by the parasite surface molecules LPG and gp63 [14, 75] involving PKC inactivation [15]. Interestingly, it was later shown that LPG of *L. donovani *promastigotes is able to block NADPH oxidase assembly at the phagosome membrane without interfering with p47(phox) phosphorylation and its ability to form complexes with p67(phox) [76]. *L. donovani *amastigotes, on the other hand, were shown to effectively block superoxide release through inhibiting the phosphorylation of the NADPH oxidase component p47(phox), leading to defective recruitment of p47(phox) and p67(phox) to the phagosome [77]. The inhibition of p47(phox) phosphorylation could be a result of the previously reported ability of *Leishmania *amastigotes to inhibit PKC activity [15], which is reported to be required for p47(phox) phosphorylation [78]. Downregulation of ROIs seems also to be modulated by ERK as inhibitor of ERK decreased ROIs production, increasing the killing of *L. amazonensis* amastigote, however this mechanism is not applied to all species as *L. major* still survives [79].

In addition to those nitrous and oxygen derivatives, IL-1 and TNF-*α* have been correlated with antimicrobial activities against bacteria and parasites *in vitro* and *in vivo *[80–83], and IL-12 is well known for its ability to promote Th1 differentiation and to activate NK cells [84]. In regard to IL-1 and TNF-*α*, it has been shown that these molecules are not produced upon a 12 h *in vitro *infection of human monocytes with *L. donovani *amastigotes [85]. Interestingly, preinfection of those cells diminished LPS-mediated IL-1 production, but not IL-1 m-RNA, suggesting inhibition at the translational level [85]. Another study showed that preincubation of human monocytes with purified LPG was able to cause inhibition of LPS-mediated IL-1*β* secretion [86]. The role of LPG in IL-1*β* inhibition was later shown to involve LPG ability to inhibit IL-1*β* gene transcription in a manner dependent on the nucleotide region −310 to −57 of the promoter region [87]. This inhibitory effect of LPG on IL-1*β* gene transcription was suggested to involve an inhibition of the binding of an activation factor or an induction of an unknown transcription repressor [87]. Interestingly, a study by our laboratory revealed that SHP-1 deficient mice infected with *L. major *produced significantly higher amounts of IL-1 and TNF-*α* compared to their littermates [61], suggesting that *Leishmania*-induced SHP-1 activity could play a pivotal role in the attenuation of the inflammatory response repressing the proinflammatory cytokines production.

IL-12 is another key cytokine inhibited by *Leishmania*. This inhibitory effect is necessary for parasite survival given the established role of this molecule in driving Th1 differentiation and production of IFN-*γ* by T cells and NK cells, which in turn can activate M*∅*s to kill the parasite. It has been reported that infection of BMDMs with promastigotes of *L. major *or *L. donovani* fails to induce IL-12 production, both following infection alone and upon subsequent LPS or heat-killed bacterial stimulation of M*∅*s [88]. Similar observations were seen when murine M*∅*s were infected with amastigotes of *L. major *and *L. mexicana* [89]. Furthermore, incubation of activated murine M*∅*s with LPG led to the inhibition of IL-12 production by these cells, with the inhibition occurring at the transcriptional level [90]. The mechanism by which IL-12 is inhibited by *Leishmania* remains not fully understood. Roles for the M*∅* CR3 [91] and Fc-*γ*R [92] have been proposed. Recently, we have reported a very interesting mechanism whereby *Leishmania* can inhibit LPS-mediated proinflammatory functions such as IL-12 and TNF-*α* production. We showed that *Leishmania*-induced SHP-1 is able to bind to an evolutionarily conserved immunoreceptor tyrosine-based inhibitory motif (ITIM)-like motif (which we renamed kinase tyrosine-based inhibitory motif (KTIM)) found in the kinase domain of IL-1 receptor-associated kinase 1 (IRAK-1), causing its inactivation. SHP-1-bound IRAK-1 is no longer able to detach from Myeloid differentiation factor 88 (MyD88) to bind TNF receptor-associated factor 6 (TRAF6) and activate downstream signalling pathways, therefore explaining in part how the parasite is able to block LPS-mediated MyD88-dependent proinflammatory functions in host macrophages [93].

One remarkable tactic the parasite utilizes to subvert the immune response is its ability to inhibit IFN-*γ*-induced MHC class II expression in infected M*∅*s. Indeed, *L. chagasi *and *L. donovani *were both shown to inhibit MHC II expression in response to IFN-*γ* stimulation [94–96]. Surprisingly, M*∅*s infected with *L. major *or *L. amazonensis *showed normal phagocytosis, Ag processing, and MHC II production, yet these cells failed to present parasitic Ags to T-cell hybridomas [97, 98]. Authors of both studies concluded that the failure to present Ags to T cells is due to the parasite's ability to interfere with the loading of Ags onto MHC II molecules. Another interesting mechanism to control Ag presentation is shown by amastigotes of *L. amazonensis* being able to internalize MHC II molecules and to degrade them using their cysteine proteinases [99].

Activation of CD4+ T cells involves a “two-signal model” whereby two signals are required to activate the T helper cell. The first signal is triggered by the binding of the T-cell receptor (TCR) to the MHC II-Ag complex on the APC, and the second is provided by the binding of CD28 or CD40L on T cells to costimulatory molecules of APCs such as those of the B7 family or CD40. Interestingly, apart from interfering with the first signal by inhibiting MHC II presentation, *Leishmania* has been demonstrated to interfere with M*∅* costimulatory signals. *L. donovani *infection was reported to block LPS-mediated B7-1 expression in infected M*∅*s [100], a mechanism that seems to be mediated by prostaglandins [101]. Furthermore, *L. major *was reported to interfere with CD40 signalling in infected M*∅*s in a p38-dependent manner [31]. This result is very interesting, especially because previous studies have established a protective role for CD40 in *Leishmania major *infections [102, 103], while others have reported that the disruption of CD40/CD40L ligation results in increased susceptibility to *L. amazonensis *infection [104]. The increased susceptibility caused by the disruption of CD40/CD40L ligation was in part due to the inhibition of iNOS expression [102, 104] and IL-12 production [105] by infected M*∅*s.

So far, we have discussed several mechanisms by which *Leishmania *can interfere with key signalling pathways involved in M*∅* activation such as the JAK/STAT pathway. We also discussed alterations that occur to signalling molecules involved in TLR signalling such as MAPKs and the TFs NF-*κ*B and AP-1. However, this does not give justice to TLR signalling, given its extremely important role in the activation of APCs to kill invading pathogens and/or activate cells of the adaptive immune system. Equally important are the strategies developed by pathogens to block TLR signalling pathways that can lead to undesirable activation of immune functions. Therefore, the last portion of this review will discuss TLR signalling and how *Leishmania* parasite deals with this important group of pathogen sensors.

## 5. Modulation of Toll-Like Receptor Signalling by *Leishmania*

TLR family members are known for their critical role in bridging the innate immune response to the adaptive one through recognizing pathogen-associated molecular patterns (PAMPs). In the light of the ongoing host-pathogen arms race, the detection of parasite PAMPs by TLRs has two main implications: first, the ability of cells of the immune system to detect parasites and eliminate them when favourable conditions are present. Second, the ability of parasites to counteract TLR detection by interfering with TLR signalling keeping immune cells in an inactive state and rendering them refractory to subsequent TLR stimulation.

One of the main parasite-derived molecules involved in TLR binding and activation is GPI-anchored proteins. *Trypanosoma cruzi*-derived GPI-anchors were shown to be detected by TLR2/TLR6 and CD14 and to activate NF-*κ*B [106, 107], while GIPLs of *T. cruzi *activated Chinese hamster ovary (CHO) cells in a TLR4/CD14-dependent manner [108]. It has been also shown that GPI-mucin of *T. cruzi* is able to activate TLR signalling on first exposure and induce tolerance to secondary TLR stimulation [109]. This was later shown to be mediated by the ability of GPI-mucin to induce the expression and activation of the serine/threonine phosphatase PP2A that acts on cellular IRAK-1, MAPKs, and I*κ*B causing their inhibition and leading to tolerance [110]. The induction of PP2A was shown to require p38 and NF-*κ*B, the very same molecules PP2A is induced to inhibit, therefore giving rise to an autoregulatory loop [110]. LPG of *Leishmania* is another GPI-anchored protein detected by TLRs. It has been shown that LPG of *L. major *directly binds to TLR2 of M*∅*s and NK cells [111, 112] and that LPG of *L. donovani *is also detected by TLR2 of activated M*∅*s [113]. Interestingly, GPI-anchors derived from *Plasmodium falciparum *merozoites can induce TNF production in human monocytes and mouse M*∅*s through interacting with TLR1/TLR2 and to a lesser extent TLR4 [114, 115]. Moreover, GPI- anchors of *Toxoplasma gondii *are detected by TLR2 and TLR4, which can thus play an important role in host defense against *T. gondii *infections [116]. 

Although less numerous than GPI-anchored ligands, non-GPI-related ligands represent an important group of parasite-related molecules detected by TLRs. An example is the *T. cruzi*-derived protein Tc52, which is able to induce proinflammatory cytokine production in DCs in a TLR2-dependent manner [117]. Other important non-GPI ligands include the DNA of *T. cruzi*, *T. brucei*, and *Babesia bovis*, which are able to activate M*∅*s and DCs [118, 119], possibly through unmethylated CpG motifs [120] detected by TLR9 [121, 122]. TLR3 was recently shown to be upregulated in IFN-*γ*-primed M*∅*s and to play a role in their leishmanicidal activity. The silencing of TLR3 led to impaired NO and TNF-*α* production in IFN-*γ*-primed M*∅*s in response to *L. donovani *infection and increased parasite survival [113]. Given that the only known ligand of TLR3 is dsRNA, the parasite component that activates TLR3 remains unclear. The authors ruled out the presence of dsRNA *Leishmania *virus infection in their parasite strain and also failed to detect natural *Leishmania*-derived double-stranded RNA structures such as rRNA or tRNA [113]. As far as apicomplexans are concerned, *Plasmodium*-derived hemozoin crystals were shown to induce proinflammatory cytokines in M*∅*s [123, 124]. Initially, TLR9 was proposed as the binding receptor of hemozoin [125], this remains controversial as it has been later shown that TLR9 activation by hemozoin is mediated by malaria DNA attached to the crystal and that the activation of TLR9 by hemozoin was abolished upon treatment with nucleases [126]. In fact, recent data from our laboratory show that the proinflammatory cytokine IL-1*β* is induced by hemozoin through the Nod-like receptor family, pyrin domain containing 3 protein (NLRP3), and the adaptor protein Asc, which lead to caspase 1 activation [127]. Concerning *Toxoplasma*, a profilin-like protein from *T. gondii *(PFTG) activates TLR11 in mouse cells [128], and heat shock proteins and partially purified preparations isolated from tachyzoites activate TLR4 and TLR2, respectively [129, 130].

The many parasite-related molecules that are detected by TLRs suggest an important role for TLR-related signalling molecules in the resistance to parasitic infections [131]. Given the fact that Th1-driving proinflammatory responses are beneficial to the host in several types of parasitic infections, it is not surprising that the activation of the MyD88-dependent pathway is crucial in the resistance to many protozoan diseases. Indeed, MyD88-deficient mice are highly susceptible to *T. cruzi *[132],* T. brucei *[122],* L. major *[133], and *T. gondii *[134] infections due to the decreased inflammatory response and the impaired production of Th1-associated cytokines such as IL-12 and IFN-*γ* in these mice. It is important to mention that MyD88-driven proinflammatory events are not always favourable to the host in the fight against protozoans. The decreased inflammatory and Th1 responses in MyD88-deficient mice were seen to improve pathology and outcome of *P. berghei* infection in mice. This suggests that *Plasmodium*, in this case, utilizes the MyD88-dependent pathway to cause tissue injury and worsen disease symptoms [135].

It is quite remarkable that the amount of susceptibility to several protozoan infections conferred by the absence of MyD88 is significantly higher than that observed when mice lacking a single TLR are used. This strongly suggests that several TLRs are simultaneously involved in the recognition of parasites, thus explaining why the loss of MyD88 can have a bigger impact on susceptibility compared to the loss of a single TLR [131]. Nevertheless, deficiency of relevant TLRs increases susceptibility to certain infections. For example, TLR9-deficient mice have higher parasitemia and mortality when infected with *T. cruzi *[121] or *T. brucei *[122]. TLR4-deficient mice are more susceptible to *L. major *infection with bigger lesion size and parasite loads compared to WT mice [136, 137], and TLR11-deficient mice are more susceptible to *T. gondii* infection manifesting increased cyst formation in the central nervous system and decreased IL-12 and IFN-*γ* production compared to WT mice [128].

The ability of TLRs to detect parasite PAMPs put together with the fact that many successful infections are associated with silent entry to target cells suggests that parasites must have evasion tactics to block TLR signalling and functions. Some of these mechanisms have been already described, while others are still to be discovered. We will hereby discuss some evasion strategies employed by *Leishmania*, *Plasmodium*, and *Toxoplasma*. 

The ability of *Leishmania *to interfere with TLR signalling components has been already discussed in this chapter under the “signalling pathways altered by *Leishmania*” section. These evasion mechanisms include the previously discussed ability of the parasite to interfere with the activation of all three MAPKs (Erk1/2, JNK, and p38) (see MAPK section) and its ability to interfere with I*κ*B, NF-*κ*B, and AP-1 (see TF section). There is also evidence that signalling through CR1 and CR3, which *Leishmania* is known to bind to, can inhibit LPS- and IFN-*γ*-induced IL-12 production through impaired STAT-1 phosphorylation [91]. A similar role for Fc-*γ*R ligation has been proposed [92, 138]. Nevertheless, very little is known about how the parasite can interfere with critical upstream proteins unique to IL-1/TLR signalling such as members of the IRAK family. Our laboratory has been interested for many years in exploring mechanisms utilized by *Leishmania* to block TLR signalling in M*∅*s, and to evaluate the role of host SHP-1 in this process (Figure 1). 

Of utmost interest, we have recently established SHP-1 as a central regulator of TLR signalling which can be exploited by *Leishmania* to inhibit IRAK-1 leading to the inability of M*∅*s to respond to a wide range of TLR ligand stimulation including LPS, favoring parasite survival [93].

Other pathogen evasion tactics include the ability of *P. falciparum *to cause infected erythrocytes to express *P. falciparum* erythrocyte membrane protein 1 (PfEMP1) which was shown to interact with the scavenger receptor CD36 on the surface of DCs [139] making the cells that phagocytose these infected erythrocytes become unresponsive to LPS stimulation, ultimately leading to defects in T-cell activation [140–142].

*T. gondii* is yet another parasite able to block LPS-mediated IL-12 and TNF-*α* production, the upregulation of costimulatory molecules, and the activation of T cells [143–146]. One way the parasite is able to do so is by activating STAT3 in IL-10-dependent and -independent manners [147, 148]. Although this *T. gondii*-induced inhibition of subsequent LPS stimulation might somehow resemble LPS tolerance in that it inhibits MAPKs like p38 [149], important differences between infection and LPS tolerance exist. Unlike LPS tolerance, *T. gondii *infection followed by LPS stimulation resulted in the activation of MKK3 and MKK6 (upstream activators of p38) and in the degradation of I*κ*B [149]. This suggests that the inactivation of p38 observed when LPS stimulation is preceded by *Toxoplasma* infection is either due to the inhibition of another p38-activating kinase such as MKK4 or is mediated by a *T. gondii-*induced MAPK phosphatase that prevents the phosphorylation-dependent activation of p38 [131]. It is interesting to note that although *T. gondii *infection followed by LPS stimulation causes I*κ*B activation, the liberated NF-*κ*B fails to translocate to the nucleus [150, 151]. Later studies suggested that the lack of NF-*κ*B translocation might actually be due to increased nuclear export of this TF rather than inhibition of nuclear import [152].

Collectively, it is clear that TLRs play a crucial role in mounting innate and adaptive immunity against invading pathogens. Alteration of TLR signalling by pathogens or by clinical drugs can play a key role in the outcome of infections. We have discussed in good detail strategies used by pathogens or by the clinic to alter TLR signalling. The activation of MyD88-dependent signalling and Th1 responses can turn out very useful in the elimination of many pathogens including *Leishmania*. However, these efforts must always be perceived with caution as exaggerated activation of inflammation can cause edema, pain, and tissue injury and in severe conditions could be deadly. In addition, certain infectious models like malaria seem to benefit from MyD88-dependent signalling and inflammation in their pathology, and thus a completely different approach should be used when trying to fight *Plasmodium*. As opposed to using TLR ligands which can worsen the disease, TLR agonists could prove clinically effective in treating malaria. Nevertheless, the effects of blocking TLR-TLR-L interactions on the ability of the immune system to fight off other pathogens that can be/become present have to be taken into serious consideration. 

## 6. Concluding Remarks

Over the last 15 years, research stemming from our laboratory and others has provided strong evidence that parasites of the *Leishmania* genus are able to establish themselves and to propagate within the mammalian host due to its ability to alter key signalling pathways, therefore interfering with the induction of critical M*∅* functions that can otherwise threaten parasite survival. Importantly, we have identified one key way *Leishmania* can do so, and this is by exploiting host negative regulatory mechanisms such as the modulation of M*∅* PTPs.
